# Operationalizing and digitizing person-centered daily functioning: a case for functionomics

**DOI:** 10.1186/s12911-024-02584-2

**Published:** 2024-06-27

**Authors:** Esther R.C. Janssen, Ilona M. Punt, Johan van Soest, Yvonne F. Heerkens, Hillegonda A. Stallinga, Huib ten Napel, Lodewijk W. van Rhijn, Barend Mons, Andre Dekker, Paul C. Willems, Nico L.U. van Meeteren

**Affiliations:** 1 Radboud Institute for Health Sciences, IQ health, Radboud university medical centre, Nijmegen, The Netherlands; 2 School of Allied Health, HAN University of Applied Sciences, Nijmegen, The Netherlands; 3 Department of Orthopedic Surgery, VieCuri Medical Centre, Tegelseweg 210, Venlo, 5912 BL The Netherlands; 4 Department of Orthopedics and Research School Caphri, Maastricht University Medical Centre+, Maastricht, The Netherlands; 5 Brightlands Institute for Smart Society (BISS), Faculty of Science and Engineering (FSE), Maastricht University, Heerlen, The Netherlands; 6 Department of Radiation Oncology (Maastro), GROW-School for Oncology, Maastricht University Medical Centre+, Maastricht, The Netherlands; 7 Dutch Institute of Allied Health Care (NPi), Amersfoort, The Netherlands; 8 Research Group Occupation & Health, HAN University of Applied Sciences, Nijmegen, The Netherlands; 9 University of Groningen, University Medical Center Groningen, Groningen, The Netherlands; 10 RIVM/ Dutch WHO-FIC Collaborating Centre, Bilthoven, The Netherlands; 11 Department of Orthopedic surgery, Utrecht University, Utrecht, The Netherlands; 12 Leiden University Medical Centre, Leiden, The Netherlands; 13 GO FAIR International Support & Coordination Office (GFISCO), Leiden, The Netherlands; 14 Top Sector Life Sciences and Health (Health~Holland), The Hague, The Netherlands; 15 Department of Anesthesiology, Erasmus Medical Centre, Rotterdam, The Netherlands

**Keywords:** Real-world data, Interoperability, Information technology, Big data, Personalized healthcare

## Abstract

An ever-increasing amount of data on a person’s daily functioning is being collected, which holds information to revolutionize person-centered healthcare. However, the full potential of data on daily functioning cannot yet be exploited as it is mostly stored in an unstructured and inaccessible manner. The integration of these data, and thereby expedited knowledge discovery, is possible by the introduction of functionomics as a complementary ‘omics’ initiative, embracing the advances in data science. Functionomics is the study of high-throughput data on a person’s daily functioning, that can be operationalized with the International Classification of Functioning, Disability and Health (ICF).

A prerequisite for making functionomics operational are the FAIR (Findable, Accessible, Interoperable, and Reusable) principles. This paper illustrates a step by step application of the FAIR principles for making functionomics data machine readable and accessible, under strictly certified conditions, in a practical example. Establishing more FAIR functionomics data repositories, analyzed using a federated data infrastructure, enables new knowledge generation to improve health and person-centered healthcare. Together, as one allied health and healthcare research community, we need to consider to take up the here proposed methods.

## Introduction

### Omics research and the definition of functionomics

An ever-increasing amount of health, healthcare and related research data on a person’s daily functioning is collected by a variety of stakeholders: people themselves, healthcare professionals and researchers, among others. By joint analysis of these data a tremendous amount of information can be derived from these data and has the potential to revolutionize person-centered prevention and healthcare, potentially improving health and life expectancy. Within the fields of oncology, radiology and genetics, computerized analysis of high-throughput data has already shown benefits for the personalization and optimization of healthcare [1–3]. These initiatives are often referred to as ‘omics’ research, where analyses of big data on genes (genomics), RNA (transcriptomics), proteins (proteomics), metabolites (metabolomics), and imaging (radiomics) are performed to advance personalized and preventive health and healthcare [4, 5]. To make this possible, ‘omics’ initiatives rely more and more on machine actionable data.

In this study we propose the integration of a new ‘omics’, namely functionomics. The current ‘omics’ research field focusses on biomedical or internal exposures, whilst functionomics can specifically contribute to eliciting interactions with personal and general external exposures (Table 1).

**Table 1:** Definitions ‘omics’ research initiatives

Feature	-omics/-ome type	Definition
Gene	Genomics	Science that studies the structure, function, evolution, and mapping of genomes and aims at characterization and quantification of genes, which direct the production of proteins with the assistance of enzymes and messenger molecules [6].
	Genome	Entirety of an organism’s hereditary information. It is encoded either in DNA or, for many types of viruses, in RNA. The genome includes both the genes and the non-coding sequences of the DNA/RNA [6].
RNA	Transcriptomics	Study of the transcriptome—the complete set of RNA transcripts that are produced by the genome, under specific circumstances or in a specific cell—using high-throughput methods, such as microarray analysis [7].
	Transcriptome	Set of all messenger RNA molecules in one cell, tissue, or organism. It includes the amount or concentration of each RNA molecule in addition to the molecular identities [6].
Protein	Proteomics	Science that studies those proteins as related to their biochemical properties and functional roles, and how their quantities, modifications, and structures change during growth and in response to internal and external stimuli [6].
	Proteome	Sum of all the proteins in a cell, tissue, or organism [6].
Metabolite	Metabolomics	Science that studies all chemical processes involving metabolites. More specifically, metabolomics is the study of chemical fingerprints that specific cellular processes establish during their activity; it is the study of all small-molecule metabolite profiles [6].
	Metabolome	Collection of all metabolites in a biological cell, tissue, organ, or organism, which are the end products of cellular processes [6].
Morphological feature	Radiomics	High-throughput extraction of quantitative features that result in the conversion of images into mineable data and the subsequent analysis of this data for decision support [8].
	Radiome	Complete set of imaging features extracted from available medical imaging in one patient [8].
**Functioning**	**Functionomics**	**Functionomics is the study of high-throughput data on daily functioning associated with health, as defined and objectified in the International Classification of Functioning, Disability and Health (ICF).** [9]
	**Functionome**	**The sum of all features of daily functioning for an individual: body functions and structures, activities and participation and those that influence functioning: environmental factors and personal factors.**

**Fig. 1:**
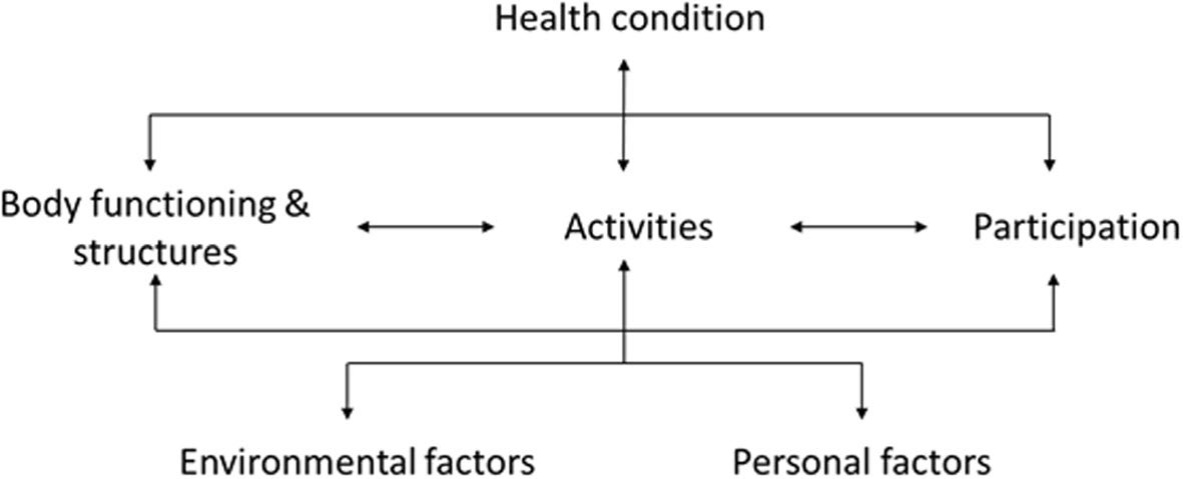
The ICF-framework

The concept of functioning and its underlying phenomena are globally described by the World Health Organization (WHO) in the International Classification of Functioning, Disability and Health (ICF) [9] (Fig. 1).

The ICF is used internationally in different types of interdisciplinary healthcare and social research settings, as well as to inform health policy development [12, 13]. Therefore the ICF is an ideal framework for providing a common format for making functionomics data machine actionable in an international setting.

**Fig. 2:**
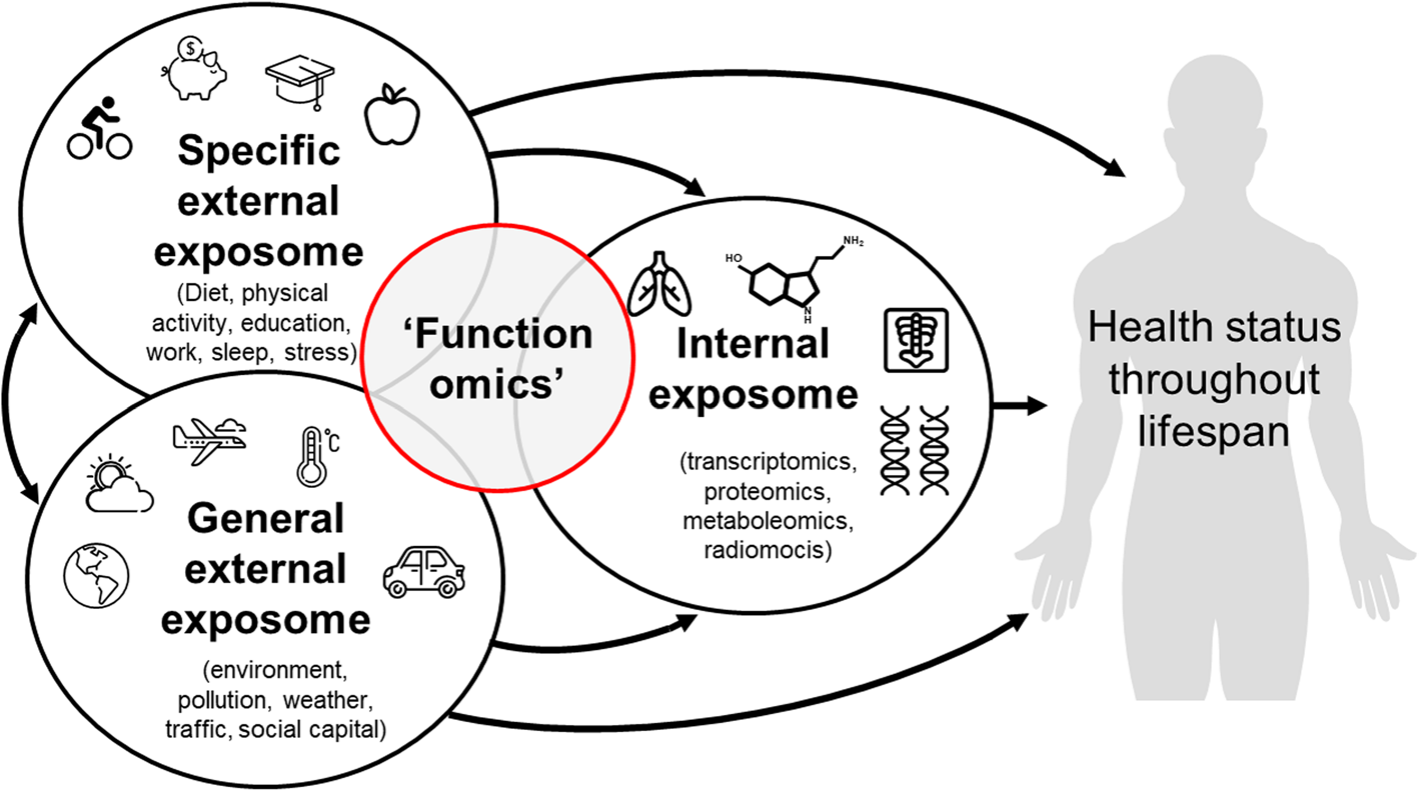
The human exposome reflects the totality of internal and external exposures within a human life cycle. Current ‘omics’ research field focus solely on biomedical or internal exposures, whilst functionomics can specifically contribute to eliciting interactions with personal and general external exposures. (adapted from Vrijheid et al. [15])

Until now the potential of functionomics data cannot yet be fully exploited, as they are usually stored in an unstructured manner in all sorts of mostly inaccessible data-silos. The lack of machine actionable data makes it difficult for people themselves as well as for outsiders (those not involved in the data collection and storage) to access, understand, analyze, interpret, and reuse these data. Imagine your own hard drive which holds all sorts of research datasets which cannot be accessed or understood by others. This prohibits joint analysis of data, causing dilution of information and loss of valuable knowledge which may result in suboptimal clinical decisions and ultimately less effective care [14]. Therefore, a transition towards the integration of functionomics as an additional ‘omics’ initiative, and at the same time embracing the advances in data science and information technology (IT), is necessary. Integrating functionomics in health, healthcare, education and research practice has an additional benefit on top of the other ‘omics’, as it provides a means to capture a more holistic view of health, rather than the limited biomedical view. Functionomics research can specifically contribute to eliciting interactions with personal and general external exposures (Fig. 2) and to broaden the scope of person-centered healthcare.

### Functionomics in the context of allied health professionals

Allied healthcare disciplines are well-positioned to pioneer a functionomics initiative, as these disciplines generate large amounts of data that can be captured within the ICF. Allied healthcare comprises a large group of health professionals, that are not physicians, with the core focus of enabling people to enjoy optimal functioning in their daily lives (e.g., physiotherapists, dieticians, speech therapists). For example: there are currently annually 3.84 million people treated by approximately 35 000 physiotherapists in the Netherlands and there are 560 000 physiotherapists in the European Union (EU) [16, 17] and 216 920 in the United States (US) [18]. If we assume that the average physiotherapist generates approximately 0.1 GB of data per patient, [19] we can estimate a data volume of roughly 375 terabytes per year in the Netherlands, 5.9 petabytes in the EU and 2.4 petabytes in the US. Translating these data into information that is actionable at the point of care and subsequently using that information to guide prognosis, diagnosis, prevention, and treatment pave the way towards more adequate and personalized physiotherapy [20, 21]. However, this huge amount of functionomics data can only be processed by machines. Subsequently, acceleration of knowledge generation can only be achieved by making data machine actionable.

### Barriers to implementation

To enable functionomics research, there are four major challenges in data collection, processing and storage that need to be addressed: 1) variability in data collection and storage strategies, 2) lack of implementation of community data standards, 3) ethical and social dilemmas like patient privacy issues, and 4) interoperability between IT systems [22]. In this paper we will focus on suggesting solutions for these challenges, where we will focus on the problem that functionomics data are currently not machine actionable as they are collected in a mostly unstructured manner and stored in inaccessible data-silos. The potential to compromise patient privacy when linking records across data silos is an additional complicating factor (challenge 3). These issues could be resolved by creating a federated functionomics data infrastructure before functionomics research can live up to its full potential and will be discussed in this paper.

### Transition from data storage to data use

A robust data infrastructure between the many data silos is a prerequisite for any ‘omics’ initiative, as it allows joint analysis of multiple data sources. Such a data infrastructure relies on usage of a ontology and data processing. Particularly, data should be transformed following the FAIR (Findable, Accessible, Interoperable, and Reusable) principles [23]. FAIR principles are internationally promoted as best practice in data management, with examples of successful application in other types of ‘omics’ initiatives [24]. FAIR principles are recommended by organizations like WHO, G20, European Commission, and European Open Science Cloud [25]. Computational ontologies and Semantic Web technologies, are strongly recommended methods to help achieve FAIR data [26]. Applying these methods will provide citizens, health professionals and researchers with machine readable data that can be analyzed via a federated data infrastructure. These concepts are currently under-utilized, as many are unfamiliar with them and what they can bring to daily life challenges up to clinical practice quests.Moreover, many of the prerequisites for making functionomics data FAIR are currently not available in this field. Combined efforts are needed to resolve these issues.

Therefore, the aim of this article is to provide a step-by-step guide on how to implement and utilize FAIR functionomics data, by proposing a method for creating an ontology based on the ICF and introducing internationally advocated concepts (FAIR principles operationalized through Semantic Web technology) for making data machine actionable. In the discussion we will address remaining issues for making data FAIR within the domain of functionomics.

## Materials and methods

In this study we used a single database example from a retrospective cohort study, to walk through the steps of creating FAIR data ready for federated analysis. The data were collected with the goal of developing a decision-support system to aid in the personalization of the perioperative care pathway by identifying which patients are at risk for worse short- and long-term outcomes [27]. This study was assessed by the local medical ethical committee AzM/UM (METC AzM/UM) and was considered not applicable to the Medical Research Involving Human Subject Act (number 2019 − 1426). In Table 2, we provide the reader with a glossary of some fundamental terms and abbreviations used in this paper.

**Table 2:** Terms and abbreviations

Computational ontology - defines a set of concepts (classes, attributes) in a specific domain, and the relationships among these concepts to explicitly represent knowledge about an application domain. Ontologies are part of the W3C standards stack for the Semantic Web, in which they are used to specify standard conceptual vocabularies in which to exchange data among systems, provide services for answering queries, publish reusable knowledge bases, and offer services to facilitate interoperability across multiple, heterogeneous systems and databases.
DOI - Digital Object Identifier; a code used to permanently and stably identify (usually digital) objects. DOIs provide a standard mechanism for retrieval of metadata about the object, and generally a means to access the data object itself.
FAIR - Findable, Accessible, Interoperable, Reusable
Federated learning - Learning from data without the data leaving the place at which it was originally stored. The algorithm visits the data storage silos and only moves aggregated results back to the sender of the algorithm.
GitHub – GitHub is a free to use cloud-based service were developers, data scientists and others can store their, mostly open, coding projects and track version of these codes.
Interoperability - the ability of data or tools from non-cooperating resources to integrate or work together with minimal effort.
OWL - Web Ontology Language; is a Semantic Web language designed to represent rich and complex knowledge about things, groups of things, and relations between things. OWL documents, known as ontologies, can be published in the World Wide Web and may refer to or be referred from other OWL ontologies.
R2RML - A language for expressing customized mappings from relational databases to RDF datasets. A mapping takes as input a logical table, i.e., a database table, a database view, or an SQL (Structured Query Language) query. A logical table is mapped to a set of RDF triples.
RDF - Resource Description Framework; a globally-accepted framework for data and knowledge representation that is intended to be read and interpreted by machines. The way RDF connects data pieces together is via triples.
RDF triple – an RDF triple consists of a ‘subject’, ‘predicate’ and ‘object’. The subject and the predicate are resources and are identified by an URI, whereas the object can be either a resource or a literal value.
REST API – is short for RESTful Application Program Interface and can be used to access and use data via the world wide web.
SPARQL - SPARQL Protocol and RDF Query Language; enables users to query (analyze) information from databases or any data source that is mapped in RDF.
Semantic Web – the Semantic Web is an extension of the current web in which information is given well-defined meaning, better enabling computers and people to work in cooperation.
URI – A Uniform Resource Identifier; is a string that provides a unique address (either on the Internet or on another private network, such as a computer filesystem or an Intranet) where a resource can be found.

### A practical example

**Fig. 3:**
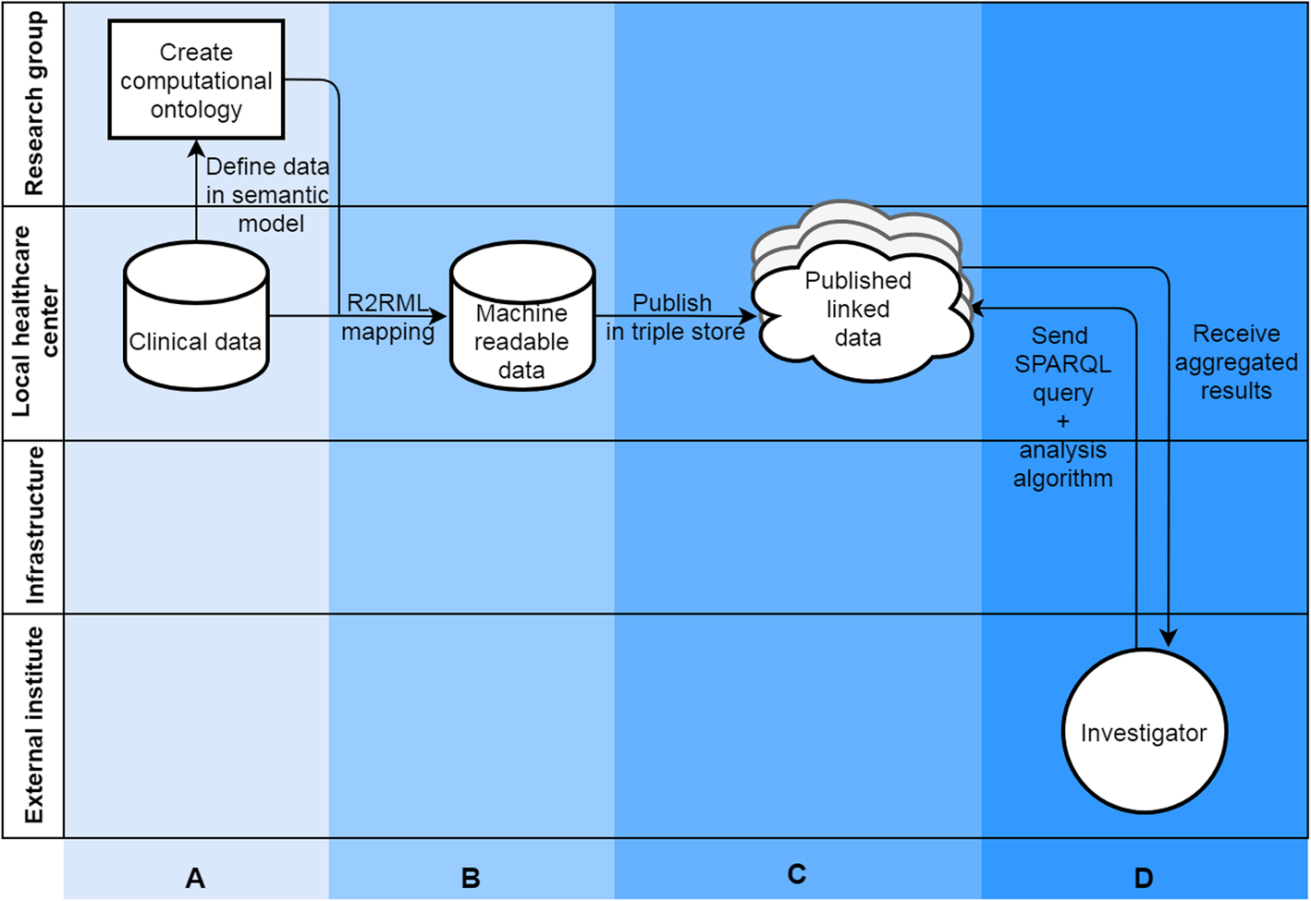
FAIRification process in a practical example. Section A: data prepping, section B: make data linkable, section C: publish FAIR data, section D: query FAIR data. If process A, B and C are repeated by different clinics multiple published linked datasets will arise that can be queried

We will describe the first steps in the methodological process to develop a FAIR functionomics database, using the above mentioned dataset, by: A) creating a computational ontology using the ICF, B) making data machine readable, C) publishing data on the Semantic Web to transform clinical data into FAIR and linked data, and D) analyzing data (queried) using a federated learning infrastructure (Fig. 3). The letters A till D in Fig. 3 are used throughout the Methods and Results sections to delimit the different steps in the process.

### Case description

Routine clinical data from 160 adult patients were collected during the perioperative care period for patients with degenerative disorders of the lumbar spine opting for fusion surgery. The database contained a set of diverse variables: patient demographic characteristics, patient-reported pain and functioning (including activities), and other clinical outcome measures (Table 3).

**Table 3:** Variables within the retrospective cohort database of patients opting for lumbar spinal fusion surgery

Variable	Value/unit	Measurement instrument
Age	Years	
Gender	Male, Female	
BMI	Weight(kilogram)/Height(in meter)^2^	
ASA-class	Class I, II, III	
Smoking status	Yes, No	
Level of educational attainment	High, Low	
Working status	Employed (fulltime), Employed (parttime), Retired, non-remunerative employment, Seeking employment	
Study identifier	Study code	
Disorder of the back	Lumbar post-laminectomy, disorder of the lumbar disc, lumbar spondylolisthesis	
Power of the muscles of the trunk	Seconds	Sorensen test
Proprioceptive function of the trunk	N correct tests	Waiter’s bow/ One leg stance/ Sitting knee extension/ Posterior pelvic tilt
Mobility of the trunk	Centimeters	Finger-floor distance
Aerobic capacity	Watt_peak_/kilogram	Steep ramp test
Pain in body part	0-100	Visual Analogue Scale
HRQOL	0-100	RAND-36 mental component and physical component subscale
ODI	0-100	Oswestry disability index
Mental health	0–21 or 0–52	Hospital Anxiety and Disability Scale or Patient Catastrophizing Scale
Lumbar spinal fusion	PLIF, TLIF, spondylodesis	
Patient encounter	Date	
N levels	Number of levels fused	
Discharge day	Date	
Postprocedural recovery status	0–30	Modified Iowa Level of Assistance Score
Postoperative complication	Yes/no	

*Abbreviations:* *ASA *American society of anesthesiology, *BMI *Body mass index, *HRQOL *Health related quality of life, *PLIF *Posterior interbody fusion, *TLIF *Transforaminal interbody fusion

### Ontology

An ontology was formulated (column A, Fig. 3) to make the data from case study interoperable. The created ontology only provides classes for concepts in our used case. It should be viewed as an example of how the allied health research community can approach building a functionomics ontology. In an ontology, a research field agrees on formal definitions of the terms in the domain and relations among them and are expressed in machine readable language [28]. A machine readable language means that computers can easily find, ‘read’ and understand data, without manual intervention. In our study, we used the open access Protégé (Stanford University, Stanford, CA, USA) software, which incorporates current standards for developing machine readable ontologies: Resource Description Framework Schema (RDFS) and the Web Ontology Language (OWL). Herein we combined terms from existing terminologies in the biomedical field to give universally agreed-upon definitions and structure to our dataset: SNOMED-CT, and Units of Measurement Ontology (UO). The ICF was used as an upper level class structure for our ontology. We added classes from SNOMED-CT and UO to define specific concepts that were available in these ontologies for variables in our dataset (e.g., age, sex). For biopsychosocial variables that could not be defined using the existing ontologies, we formulated a new class. The basic idea of this mapping process was to link each data structure (row, columns and values) within the database to its corresponding component (concept, property, relationship). The way variables are interlinked was defined within the ontology and was based on clinical expertise and understanding of these relationships by the authors. These components were developed using feedback loops with experts in the field of lumbar spinal fusion (LSF), perioperative care and the ICF. The reader should keep in mind that this is only an example, ontologies are flexible and can easily incorporate new variables and relationships or adjust existing variables/relationships. Ideally an ontology should be based on international community standards and consensus.

### Semantic web technologies

Semantic Web technologies are an extension of the World Wide Web (WWW) and provide people with a means of publishing and storing data on the Web. Within the Semantic Web, data are described in triples, based on the Resource Description Framework (RDF; column B, Fig. 3). A triple consists of three components, namely: a subject, a predicate and an object. Each of these components has a semantic definition, defined within the ontology. These three components from the defined ontology are combined to make a triple, for example see Table 4:

**Table 4:** Example of a RDF based triple

RDF triple	Subject	Predicate	Object
In the dataset	Patient_1	is diagnosed with	lumbar spondylolisthesis
Semantic triple	Patient_1	is_diagnosed_with_patient_1_diangosis	patient_1_diangosis
Definition from vocabulary	rdf: type SCTID:116154003	patient_1_FUN	rdf: type SCTID:32117100119102

In a relational database, all variables within a two-dimensional table (e.g., csv file, excel file, SPSS file) have a relation to each other, which needs to be defined in the process of making data machine readable. In this study we used R2RML descriptions to transform our data into RDF triples using the Ontop software package. Once in the dataset all data were transformed into RDF triples, the triples were stored on a web platform called GraphDB (Ontotext, Sofia, Bulgaria) running on the hospitals’ intranet (column C, Fig. 3). We checked the triple mapping using the visual graph interface of GraphDB. The intranet is a private part of the WWW, accessible only to employees of the hospital. The GraphDB instance held the RDF triples and hosts a REST API to receive requests to query the data hosted in the GraphDB instance. The universal language that can be used to query data transformed into RDF triples is SPARQL.

The Personal Health Train (PHT) [10] federated infrastructure allows a researcher or other external parties to perform analyses on data from multiple GraphDB instances or data silos without physically having access to the data (column D, Fig. 3). Through the REST API in the PHT infrastructure a researcher can send their analysis to one or more data stations communicating with a central PHT server. Subsequently the analysis is performed locally in data stations (e.g. hospitals, physiotherapy practices) and only aggregated results are sent back to the researcher via the same infrastructure. This infrastructure can be utilized to send all different types of data analyses – queries and algorithms – to the data stations, like quality assessment, prediction modelling or effectiveness calculations. A SPARQL query and algorithm for performing a simple count of gender was written and performed via the federated infrastructure.

To assess the FAIRness of the data (e.g., the degree to which the digital resource adheres to the FAIR data principles) the data was analyzed using the FAIRMetrics [11]. We used the standardized FAIR maturity indicators manual assessment, which assesses Findability, Accessibility, Interoperability and Reusability of the resource using thirtyfour indicators.

## Results

As this paper aimed to provide guidance on how to implement functionomics in clinical practice, a step-by-step tutorial of the described results was created in our GitHub repository: https://github.com/ERCJanssen/Functionomics. Readers can use this tutorial including dummy data similar to the real dataset to recreate the same steps themselves.

### Ontology

**Fig. 4:**
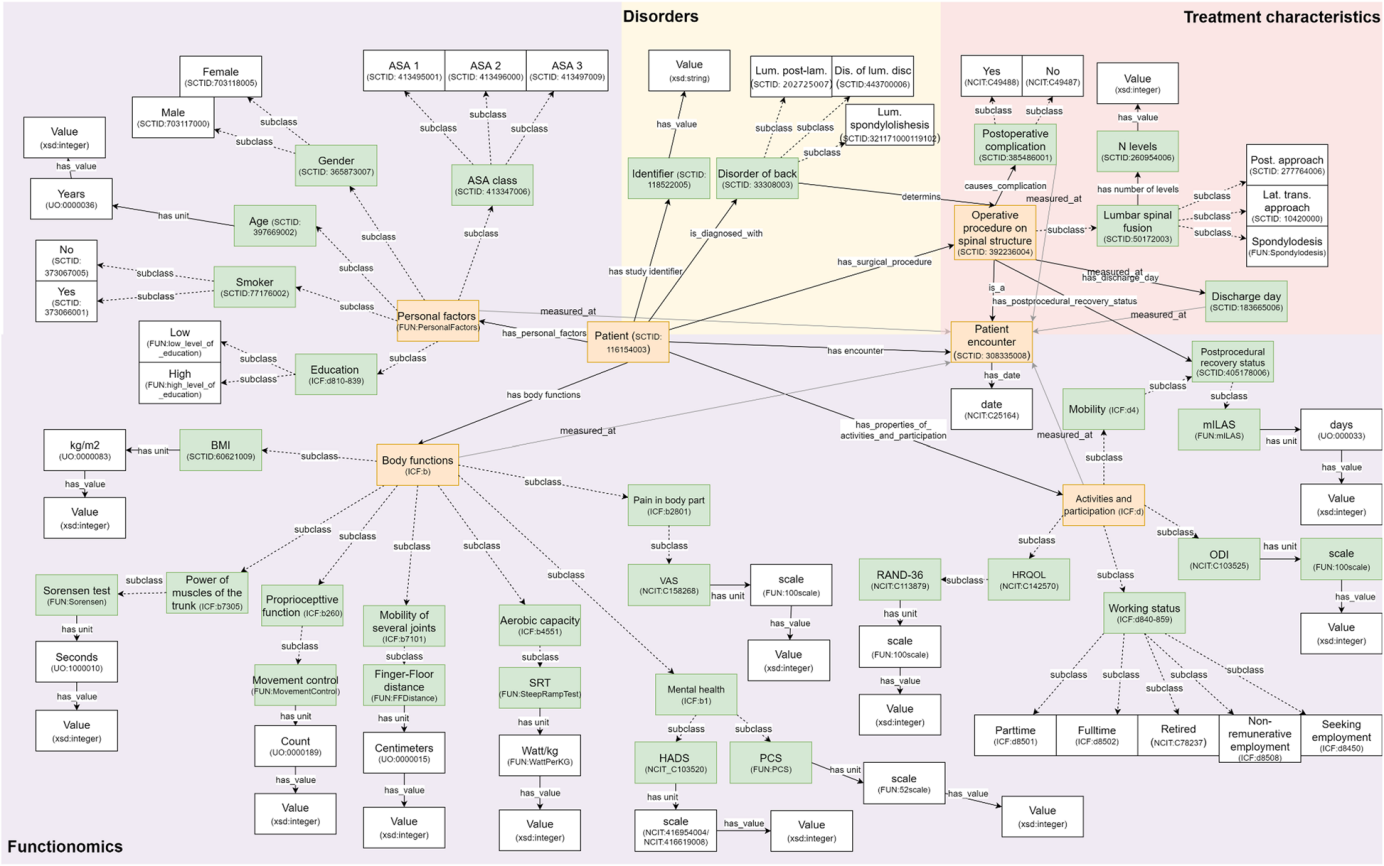
Basic concepts and relationships within the example dataset, defined within different existing ontologies. *Abbreviations:* *ASA *American society of anesthesiology, *BMI *Body mass index, *DIS* Disease, *HADS *Hospital anxiety and depression scale, *HRQOL *Health related quality of life, *KG *Kilograms, *LAM* Laminectomy, *LAT* Lateral, *LUM* Lumbar, *mILAS *Modified iowa level of assistance scale, *ODI *Oswestry disability index, *PCS *Pain catastrophizing scale, *POST *Posterior, *SRT *Steep ramp test, *TRANS *Transversal, *VAS *Visual analogue scale

We developed an ontology describing basic concepts, relationships and properties within the preoperative context of a patient deciding forLSF (column A, Fig. 3).The ICF was used as the upper level hierarchy of classes for this ontology, containing 1,596 classes. We added ‘new’ lower level concepts to the ICF structure when these concepts, defining the variables in our dataset, were not available in the ICF. We mapped all variables from our dataset as concepts in the ontology reusing concepts from well-known published ontologies, wherever possible (e.g., SNOMED CT and UO).If no appropriate concepts or relationships were available in existing ontologies, which was often the case for data about a patient’s daily functioning, new concepts were added. In total we added 42 classes and 10 predicates to the ontology. From this process we can see that many of these ‘new’ concepts about a patient’s daily functioning can appropriately be mapped to the ICF hierarchy. This ontology was published on Github (Fig. 4). The ontology can be (re)used and fine-tuned by others to fit their data on a person’s daily functioning.

### Using semantic web technology

**Fig. 5:**
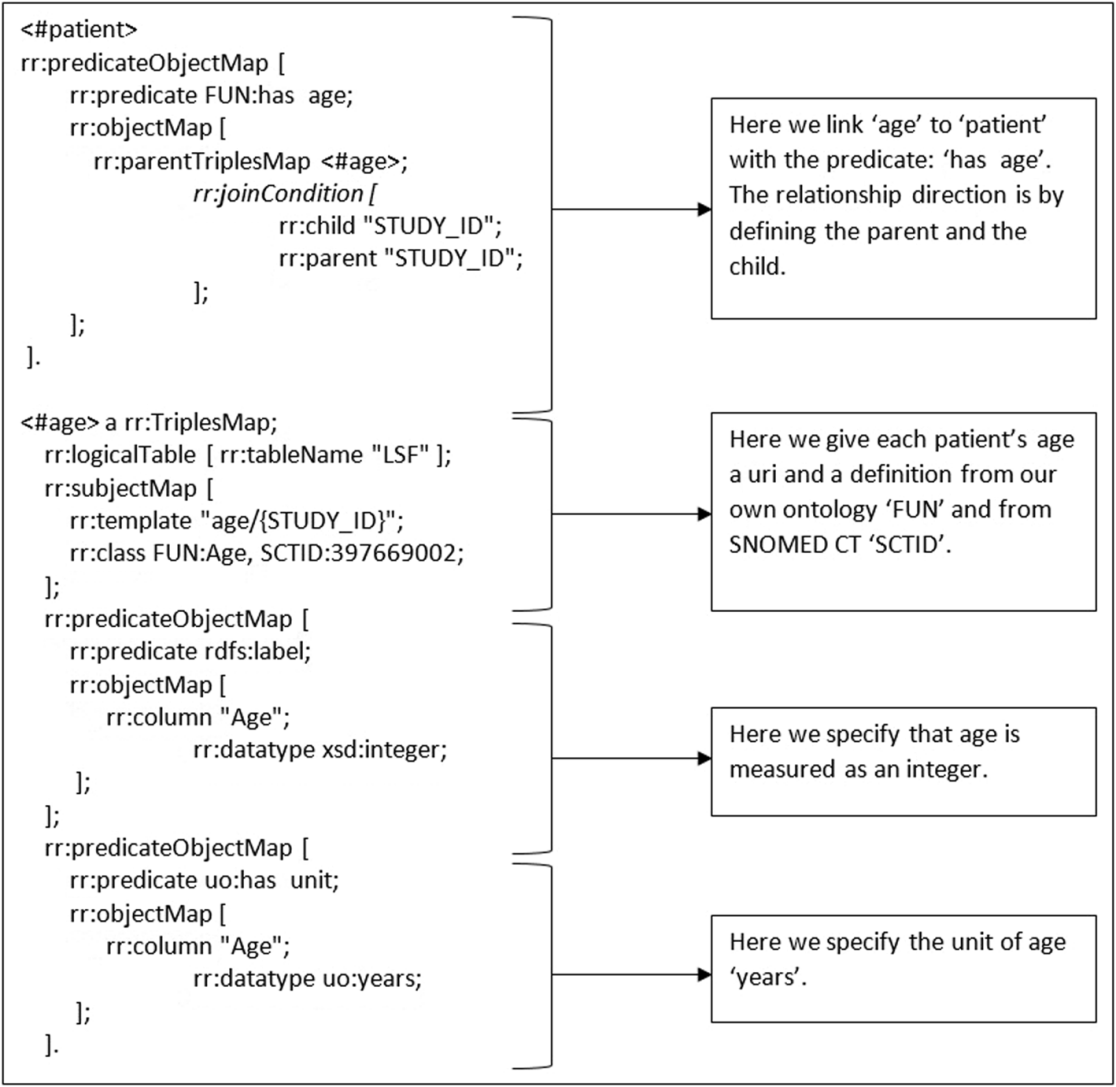
Example of an R2RML mapping to FAIRify functionomics data

To transform the .csv dataset into machine readable data (RDF triples) we made an R2RML script (column B, Fig. 3). This script reads the .csv file, using the previously created ontology, and is translated it into 74 triples. An example of the mapping file is shown in Fig. 5. The full mapping can be found on GitHub.

The RDF mapping and data were published in a GraphDB instance on a local server, which linked the data repository to the web (column C, Fig. 3). From this point on, data could be analyzed by external parties by linking to the GraphDB instance via the PHT infrastructure (column D, Fig. 3).The PHT infrastructure allows the researcher to perform analysis without having to physically collect the data in a central server [29]. To perform such an action we linked two computers via internet in a password secured infrastructure to prevent unauthorized access to the data. A researcher then sent the example SPARQL query and algorithm to our GraphDB instance via the PHT infrastructure from their own computer. The results of this query were calculated locally in our local GraphDB instance. Subsequently, the aggregated results – frequencies of gender - of this simple query were sent back the researcher via the PHT infrastructure: N females = 101, N males = 59.

FAIRness of the data is described in GitHub repository. The main focus of this example was on the interoperability part of the FAIR principles, as such the scoring for FAIRness metrics on policies is low.From the applicable indicators we scored 7/8 for findability, 2/3 for accessibility, 7/7 for interoperability and 1/4 for reusability.

## Discussion

Recent history shows the usefulness of big data analysis in personalizing healthcare through ‘omics’ research in many medical fields [5, 30, 31]. In our practical example we redefined functionomics to include data on daily functioning of a person and showed how it can be operationalized and used, here in a clinical setting. A functionomics ontology for the specific setting and population of the example was created, based on the ICF. Both biomedical and psychosocial data were transformed into a machine readable language (RDF) and published on the web (Graph DB instance). Next, these data were queried (using SPARQL) and gender counts were generated via an analytic algorithm. This paper and the tutorial in the accompanying GitHub repository enables others to familiarize themselves with the proposed approach, establish their own functionomics data station and send all different types of analyses to these stations. Using this approach we will be able to create a network of linked FAIR functionomics datasets.

From its conception many scientific breakthroughs have been established through ‘omics’ research. For example, in the recent years, radiomics has made a serious impact on personalization of radiotherapy, due to firm investment in available IT and statistical solutions [2]. This has resulted in multiple scientific and clinical advancements; for example, an internationally validated prediction model for cancer survival has been developed and new knowledge on tumor phenotypes has been generated [31, 32]. However, when considering the whole human exposome, major concepts are often not included in these ‘omics’ research types: the specific and general external exposome, and a considerable amount of the biopsychosocial aspect of the internal exposome. To further improve health and healthcare research, all elements of the human exposome should be included, informed by a biopsychosocial perspective.

In our practical example, we suggested how to operationalize this transition towards functionomics by using the ICF for the development of an appropriate ontology. The ICF is an international framework and terminology often used by allied healthcare professions to describe and organize data on a patient’s daily functioning. However, the transition from the ICF to a functionomics ontology requires to solve some major gaps in knowledge established in our study. Firstly, a classification of personal factors is lacking in the current ICF class hierarchy and – although different articles are published with preliminary lists - the WHO has decided to refrain from a classification of personal factors in the near future [9]. Secondly, no predicates were available in the ICF to establish relationships between classes. Thirdly, some concepts are hard to map within the current ICF class hierarchy, as they involve multiple ICF classes. In the community there is disagreement on methods of measuring functioning and how to map different concepts to the ICF [33]. Mapping the perception of one’s quality of life, for example, has led to some discussion about its position in the class hierarchy in our practical example as well as in other research, [34] even when applying the linking rules of the ICF [35]. Without consensus on this issue, it will remain difficult to make functionomics data FAIR. Therefore, we propose to address these gaps in knowledge in an international and interdisciplinary collaboration, to enable structured capture of real-world functionomics data. By addressing these issues, we can make functionomics operational, firstly in datasets and field examples, and step by step around the globe.

Making data FAIR has scientific value with a tremendous impact on population health, healthcare and the economy. The cost of not making data FAIR comes at a high price; annually around €100 billion is lost due to missed innovation opportunities [36]. We invest large amounts of time and effort in data capturing, but these data are only operable for single-use purposes, as they are mostly captured in an – when considering a global scale – unstructured and inaccessible manner. The FAIR principles, operationalized in Semantic Web Technology, guide the development of a global infrastructure and tooling to make all health and research data optimally reusable for machines and people alike resulting in the internet of FAIR data and services, where data, far more divergent than just health and research, can be found, accessed, and (re)used by anyone [25]. Accomplishing this will revolutionize the scientific and societal value of this data.

A major advantage of applying Semantic Web technologies and building a functionomics ontology is the ability to link different silos of data and concurrently to query them. In our example an external researcher was able to query our data without it leaving the data silo based in the hospital. Moreover, the researcher only received the aggregated results and not the individual patient data, meaning it is privacy preserving. Applying these techniques can help to solve the issues of physical data integration.

Ultimately, this approach could lead to ‘digital twins’, where one would be in the possession of very detailed biopsychosocial information of a person over time and relate them to similar persons who already underwent diagnostic, prophylactic and/or therapeutic interventions for their health challenges and very accurately predict their health outcomes [37].

### Possible barriers for implementation of functionomics

An important issue that we have not addressed in this paper is unstructured, free text, data describing a person’s functioning. Often data on functioning are not collected in a structured manner, as from our example. Concepts of functioning, including the influencing contextual factors (personal and environmental factors), are hard to capture in a cohesive whole using measurement tools. There are two ways we can deal with this issue. The first one is investing in making functionomics data more structured, for example by creating new validated measurement tools and implementing these tools in standard clinical and research practice. However, as mentioned above, data on functioning is very context sensitive, using measurement tools we may lose this context and may not accurately present the patient’s perspective [38]. The second approach is to apply free text mining, like natural language processing (NLP), to extract meaningful concepts from the free text and convert them to structured formats.

Our current science landscape does not promote data and knowledge sharing [39]. This issue is inherent to putting great value on impact factors, publication numbers and grant acquisition. A major worry for many is that when data are shared too early, others will foreshadow their work [40]. Another issue is the analysis of privacy sensitive healthcare data, stored at many different locations. Functionomics data are often collected on the same person by different healthcare providers, social organizations or even by people themselves. Combining these privacy sensitive data repositories for functionomics research requires a privacy-preserving approach. By using federated learning techniques we could largely solve this issue, as it enables local analysis of data with only aggregated results leaving the place of storage, through privacy-by-design. Still, it is obligatory to gain informed consent of any individual to use their healthcare data for research purposes. This would not be feasible in the proposed system, as different types of queries could be sent to the data silo on a daily basis. A tiered informed consent may be a viable solution. Here people grant permission for the (research) purposes of their choice, but not for all [41].

Another thing to keep in mind is that FAIR is not equal to Open: The ‘A’ in FAIR stands for ‘Accessible under well-defined conditions’ [42]. Even when publishing data on the Semantic Web it is still stored locally on a ‘private’ network. The ‘owner’ of the data can still control who gets access to them, in our case through the PHT network, for example by requiring password authentication and authorization. In contrast, opening up data (Open Access) yields most benefits, as it provides researchers access to large amounts of data to analyze.

### The next steps in functionomics

Big data analysis is not only a way to improve the robustness of science today, but can drive new scientific discovery of tomorrow. The analysis of big data on functionomics will give valuable insight in how to move forward in personalizing healthcare. For this, an internationally accepted functionomics ontology should be built, capturing all relevant data from the ICF, open access mapping scripts, a trustworthy data infrastructure and international agreements on data usage policies. Therefore, we call to action to all stakeholders in functionomics to contribute to a new ontology and participate in making their (own) data more FAIR.

## Conclusion

In this study functionomics as the study of high-throughput data on daily functioning, as defined and objectified in the ICF, is introduced. Functionomics research can have great benefits for health and person-centered healthcare, thus improving health *of* people and *with* people. Investments, by an international community in the domain of functionomics, in the proposed IT solutions for big data analysis - FAIR principles through Semantic Web technologies - are necessary to achieve this. Together, as one united health and care (research) community, we need to make serious efforts to take up the proposed methods.

## Data Availability

The data that support the findings of this study are not openly available due to reasons of sensitivity. Data are, however, available from the authors upon reasonable request and with permission from the local medical ethical committee (METC UM/AzM). Data are located in controlled access data storage at Maastricht UMC+.
